# A Rule-Based Prognostic Model for Type 1 Diabetes by Identifying and Synthesizing Baseline Profile Patterns

**DOI:** 10.1371/journal.pone.0091095

**Published:** 2014-06-13

**Authors:** Ying Lin, Xiaoning Qian, Jeffrey Krischer, Kendra Vehik, Hye-Seung Lee, Shuai Huang

**Affiliations:** 1 Department of Industrial and Management Systems Engineering, University of South Florida, Tampa, Florida, United States of America; 2 Department of Electrical and Computer Engineering, Texas A&M University, College Station, Texas, United States of America; 3 Department of Pediatrics, College of Medicine, University of South Florida, Tampa, Florida, United States of America; University of Catanzaro Magna Graecia, Italy

## Abstract

**Objective:**

To identify the risk-predictive baseline profile patterns of demographic, genetic, immunologic, and metabolic markers and synthesize these patterns for risk prediction.

**Research Design and Methods:**

RuleFit is used to identify the risk-predictive baseline profile patterns of demographic, immunologic, and metabolic markers, using 356 subjects who were randomized into the control arm of the prospective Diabetes Prevention Trial-Type 1 (DPT-1) study. A novel latent trait model is developed to synthesize these baseline profile patterns for disease risk prediction. The primary outcome was Type 1 Diabetes (T1D) onset.

**Results:**

We identified ten baseline profile patterns that were significantly predictive to the disease onset. Using these ten baseline profile patterns, a risk prediction model was built based on the latent trait model, which produced superior prediction performance over existing risk score models for T1D.

**Conclusion:**

Our results demonstrated that the underlying disease progression process of T1D can be detected through some risk-predictive patterns of demographic, immunologic, and metabolic markers. A synthesis of these patterns provided accurate prediction of disease onset, leading to more cost-effective design of prevention trials of T1D in the future.

## Introduction

Type1 Diabetes (T1D) is an autoimmune disorder that has a diverse pathogenesis, clinical phenotype, and outcome [1]. It is associated with a progressive immune-mediated loss of insulin-secreting islet cells, leaving a trail marked by characteristic immunologic and metabolic signs that provide predictive markers of disease [2], such as the 2 hour glucose and C-peptide markers. The increasing understanding of the immune pathogenesis of T1D has led to the possibility that preventive interventions could delay or prevent its occurrence. Some prevention trials, such as the European Nicotinamide Diabetes Intervention Trial and the Diabetes Prevention Trial-Type 1 (DPT-1), have been launched to test interventions in individuals with autoimmune pre-diabetes [3]–[5]. They have shown that individuals with certain degree of risk can be recruited through the assessment of relatives of T1D patients by autoantibody and metabolic testing.

Despite the possibility of predicting T1D on the basis of genetic, immunologic, and metabolic markers [2], [6]–[8], there are still a substantial proportion of those classified as high risk who do not progress to clinical diabetes within five years of detection. Therefore, a number of studies have been conducted to refine the prediction of T1D to improve accuracy and efficiency, by developing risk score models using all the potential risk markers [6]–[8]. For example, a risk score is developed in [6], which was shown to be helpful for the prediction of T1D in relatives of patients who are autoantibody-positive. The Cox proportional hazards regression model (CPH) has been the most commonly used model in the past. The CPH model is appealing due to its mathematical simplicity and computational convenience, in which the baseline survival function does not need to be modeled explicitly in the model training. However, the CPH model was essentially designed to measure the effects of covariates on changing the hazard function but not modeling individual patient risk. In other words, the CPH model is not suitable to predict an individual's risk since the hazard function is incomplete without the baseline survival function. Although it is possible to fit a non-parametric baseline survival function after the CPH model is trained on data, its applicability is still limited by its proportional hazards assumption. As a result, existing risk-scores models [6]–[8] are only capable of stratifying subjects into different risk levels, rather than assessing individual's risk and predicting the disease onset.

The difficulty in developing a risk score model comes from the heterogeneity of T1D and the complex interplay among diverse risk factors. It is known that within the same genetically predisposed children, pre-diabetic subjects may have very heterogeneous characteristics with regards to the expected rates of seroconversion to islet autoantibody positivity as well as diagnosis of T1D [9]. Existing research on T1D risk model development has shown that a combination of multiple markers may produce high predictive accuracy for early disease prediction [6]–[8], [10].

In this paper, we seek to explore whether genetic, immunologic, and metabolic markers tend to occur in a predictable pattern that might be captured by statistical analysis and could therefore be used to dissect out individual's disease risk. We use the powerful rule-discovery algorithm, the RuleFit [11], to systematically explore the possible risk-predictive baseline profile patterns, defined as “rules”. Then, we develop a novel latent trait model to model the association of the rules with the underlying disease risk and further link the disease risk with the clinical outcome, forming the basis for risk estimation and outcome prediction. Our main contributions are: 1) we have identified novel and meaningful risk-predictive rules from the DPT-1 participants, which may lead to a better understanding of disease progression and high prediction accuracy for T1D; 2) we have developed a novel latent trait model which is capable of synthesizing the rules for prognosis, and, by extracting the item information curves (IIC) from the latent trait model, it is also possible to investigate the distribution of prognostic power of each rule over the spectrum of disease severity, revealing which marker measurements produce superior prognostics for a given cohort. Such a prognostic model with consideration of baseline profile patterns that involve interactions between markers, will enable more homogeneous risk grouping as well as identification of intermediate checkpoints and surrogate end points, which may lead to novel cost-effective screening strategies for future clinical trial design in preventing T1D.

## Methods

### 1. Data

The Diabetes Prevention Trial-Type 1 (DPT-1) was one of the largest randomized, prospective studies in North America from 1995 to 2003, with the objective to determine if T1D can be prevented or delayed by a preclinical intervention of oral insulin intake or low-dose insulin injections. The DPT-1 consists of two separate trials, one of oral insulin (to induce oral tolerance) and the other of parenteral insulin with daily subcutaneous low-dose insulin and annual intravenous insulin. A total of 103,391 first- and second-degree non-diabetic relatives of individuals were screened for ICA-positive subjects. The 3,483 relatives positive for islet-cell antibodies (ICA) were then staged to quantify the projected five-year risk of diabetes [12]. Of those, 372 subjects whose five-year risk was considered to be 25% to 50% with normal oral glucose tolerance test (OGTT) were entered into the oral insulin trial; 339 subjects whose five-year risk was considered to be 50% to 70% with abnormal OGTT or loss of FPIR to an intravenous glucose tolerance test (IVGTT) were entered into the parenteral trial. The criteria for eligibility can be found in [12]. All subjects (and/or their parents) signed a written consent form approved by the participating study center's human subjects committee.

To study the natural history of the disease, only the subjects who were randomized to the control arms of the studies were used in this analysis (186 from oral trial placebo arm and 170 from parenteral trial observation arm). We plan to focus on demographic, immunologic, and metabolic markers. Specifically, we will use the titer values for different autoantibodies for assessment, including ICA, IAA, GAD, ICA512, and MIAA (micro-insulin autoantibodies). For metabolic indices, we have fasting glucose, glycated hemoglobin (HbA1c), fasting insulin, first-phase insulin response (FPIR) from IVGTTs, and Homeostasis model assessment of insulin resistance (HOMA-IR). From OGTTs, in addition to 2-hour glucose and fasting glucose, we have collected blood samples for C-peptide measurements in the fasting state and then 30, 60, 90, and 120 minutes after oral glucose. We also have computed peak C-peptide as the maximum point of all measurements and AUC (area under curve) C-peptide using the trapezoid rule. Furthermore, we also include age and Body Mass Index (BMI), which have been typically included as covariates in most of the existing risk models for T1D. The baseline statistics of the study subjects are presented in Table 1.

**Table 1:** Baseline statistics of the study subjects.

	Oral Insulin Trial N = 186	Parenteral Insulin Trial N = 170
IDDM (%)	53(28%)	70(41%)
Age -year (mean)	12.30(8.60)	15.34(9.92)
BMI Z-score (median) *	−0.90(−2.35−0.38)	−1.48(−3.04−0.01)
Race *n(%)*		
White	163(89.07%)	128(95.18%)
African American	2(1.09%)	1(0.60%)
Hispanic	14(7.65%)	5(3.01%)
Other	7(3.76%)	6(3.53%)
Gender *n(%)*		
Male	105(56.45%)	89(52.35%)
Female	81(43.55%)	81(47.65%)
Relationship to patient w/diabetes *n(%)*		
Sibling	108(58.06%)	113(66.47%)
Offspring	53(28.49%)	39(22.94%)
Parent	7(3.76%)	5(2.94%)
Second Degree	18(9.68%)	13(7.65%)
**HLA genotype:**		
Priamryhaptype		
0101/0501	27(14.59)	14(8.24)
0102/0604	10(5.41%)	16(9.41%)
0201/0201	12(6.49%)	10(5.88%)
0301/0301	19(10.27%)	11(6.47%)
0301/0302	77(41.62%)	75(44.12%)
0501/0201	16(8.65%)	17(10.00%)
Other	25(13.44%)	10(5.88%)
Secondayhaptype		
0301/0301	8(4.32%)	12(7.06)
0301/0302	71(38.38%)	45(26.47%)
0501/0201	69(37.30%)	81(47.65%)
0501/0301	15(8.11)	15(8.82)
Other	23(12.37%)	17(10.00%)
**Immunological factors:**		
ICA titer (JDF Units**) (median)	80.00(40.00–160.00)	160.00(40.00–320.00)
IAA titer (nU/ml) (median)	192.30(83.30–435.70)	109.25(26.70–295.34)
ICA512 (median)	0.033(0.006–0.677)	0.081(0.003–0.645)
GAD65(median)	0.204(0.027–0.677)	0.322(0.024–0.738)
**Metabolic factors:**		
Fasting Glucose (mmol/L)- IVGTT	4.84(0.51)	4.94(0.49)
Fasting Insulin (mU/L)-IVGTT	15.41(9.68)	12.01(7.76)
FPIR (ul/ml)-IVGTT	158.88(99.16)	72.80(37.10)
HOMA-R-IVGTT	3.39(2.35)	2.69(1.84)
FPIR/HOMA-R-IVGTT	55.64(33.13)	32.90(17.09)
Fasting Glucose (mg/dL)-OGTT	86.20(7.78)	89.22(9.58)
Two-hour Glucose (mg/dL)-OGTT	105.74(19.57)	122.28(31.59)
Peak C-Peptide (nmol/L)-OGTT	5.44(2.19)	4.84(1.97)
AUC C-Peptide(nmol/L)-OGTT	508.70(205.79)	439.28(174.03)
HBA1C	5.33(0.34)	5.38(0.50)

Note: Data are mean (± SD), n (%), or median (Inter-quartile range).

*BMI Z-score from 2000 CDC Growth chart.

**JDF denotes Juvenile Diabetes Foundation.

### 2. Statistical methods

Let 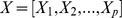
denotes the *p* pvariables corresponding to candidate risk markers introduced in Section 2.1. Our hypothesis is that there are unknown baseline profile patterns, indicating risk levels for individuals, which can be characterized as rules over these markers. As these baseline profile patterns are largely unknown and a risk estimation mechanism using these patterns is also lacking, in this paper, we propose an integrated framework of an existing rule-discovery algorithm, the RuleFit [11] and a novel latent trait model that will be developed in this paper, to fill in these gaps. Specifically, the rule-discovery algorithm can be used to discover the hidden rules that may be predictive to the disease risk. The latent trait model will be used to model the associations between the identified rules with the underlying disease risk and further estimate individual disease risk by probabilistic inference, based on these associations.

#### 2.1. Rule discovery by RuleFit

We use rules to define the baseline profile patterns, sincerules can be easily interpreted, easily handle heterogeneity and complex interaction between markers. Essentially, a rule defines the abnormal range of some markers. With the presence of an abnormality, the disease risk increases. Thus, a comprehensive set of risk-predictive rules act as a set of sensors dispersed over the whole course of disease progression, providing us the evidences for risk estimation by looking into each individual's profile of abnormalities. On the other hand, this set of rules for characterizing T1D progression is currently lacking, as the etiology of T1D is still not fully understood [13]. As traditional epidemiology studies mostly focus on studying hypotheses regarding individual risk factors, knowledge about the heterogeneity and complex interplay between risk factors that are crucial on defining the rules remains largely unknown.

We use RuleFit[11] to discover the hidden rules that may be predictive of the disease risk. RuleFit is a high-dimensional computational algorithm for rule discovery, which is capable of exhaustively searching for potential rules on a large number of candidate risk markers. It has two phases, the “rule generation phase” and “rule pruning phase”: 1) **Rule generation**: At this stage, random forest [14] is used to exhaustively search for candidate rules over the potential risk factors. Random forest is a high-dimensional rule discovery approach that extends traditional decision tree models. Specifically, a random forest estimates a number of trees, with each tree being estimated on a relatively homogenous subpopulation generated by bootstrapping the original dataset. Since each tree employs a set of rules to characterize a subpopulation, the random forest is actually a comprehensive collection of rules that are able to characterize the whole dataset. On the other hand, as a heuristic and exhaustive search approach, the random forest may produce a large number of less-predictive or redundant rules, which requires the following second step to refine the learning results. 2) **Rule pruning**: As the random forest will generate many rules that can be redundant or irrelevant to early withdrawal due to overfitting, the sparse regression model [11], [15] will be applied to select a minimum set of risk-predictive rules, by using all the potential rules as predictors and the withdrawal status as the outcome. The sparse regression model is a high-dimensional variable selection model [11], [15]. Considering each rule as a “variable”, rule pruning is essentially a variable selection problem. This problem is to selecting a subset of rules out of a pool of 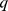
candidate rules, denoted as 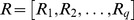, which are predictive to the output variable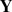. This problem is particularly challenging in high-dimensional settings where 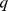
is large. Recently, the *Least Absolute Shrinkage Selection Operator* (LASSO) is proposed [15], which is a sparse linear regression model that is capable to identify a subset of relevant variables out of a huge list of candidate variables. Specifically, the formulation of LASSO is
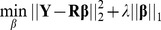



Here, the square error term,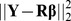, is used to measure the model fit. The L1-norm penalty term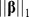, defined as the sum of the absolute values of all elements in 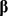, is used to measure the complexity of the regression model. The user-specified penalty parameter,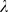, aims to achieve an optimal balance between the model fitness and model complexity – larger
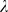
will result in sparser estimate for 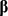. Efficient algorithms have been developed to solve the optimization problem [11], [15]. In our study, since the output variable 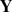, i.e., the withdrawal status, is a binary variable, the sparse logistic regression [15] is a better choice than linear regression, which can be readily implemented in the R package of RuleFit [15]. More details on RuleFit can be found in [15].

In a summary, RuleFit is computationally efficient since efficient algorithms have been developed for both Random Forest and sparse linear regression models. Since it is an integration of random forest and LASSO, it has several important parameters to be specified, including the number of trees, the complexity of the trees that is controlled by the average number of terminal nodes, and the penalty parameter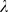. According to the extensive simulation studies performed in [15], the default parameters values for the number of trees and the average number of terminal nodes are 333 and 4, respectively. We obtained the optimal values of these three parameters using the automated cross-validation procedure in Rulefit in a manner of grid search, which are close to these default values, e.g., the number of trees and the average number of terminal nodes are 250 and 4.5, respectively. In our experiments, we have found that the RuleFit is robust to the specification of these parameters.

#### 2.2. Latent trait model

Disease risk is a latent trait that is not directly measureable. As we mentioned earlier, rules are essentially measureable evidence that are associated with the underlying disease risk. Specifically, as a rule defines the abnormal range of some markers, the satisfaction of a rule corresponds to the presence of an abnormality. It is known that some abnormalities can most likely be observed at certain progression stages, which provide us the possibility that we can infer the most possible disease risk for each subject based on this individual's profile of abnormalities, if the associations between the abnormalities with disease severity can be modeled. This statistical inference problem bears a resemblance with the classic problem in psychometrics, the inference of abilities, attitudes or personalities by gathering evidence from questionnaire responses or tests, using the latent trait theory.

**Figure 1:**
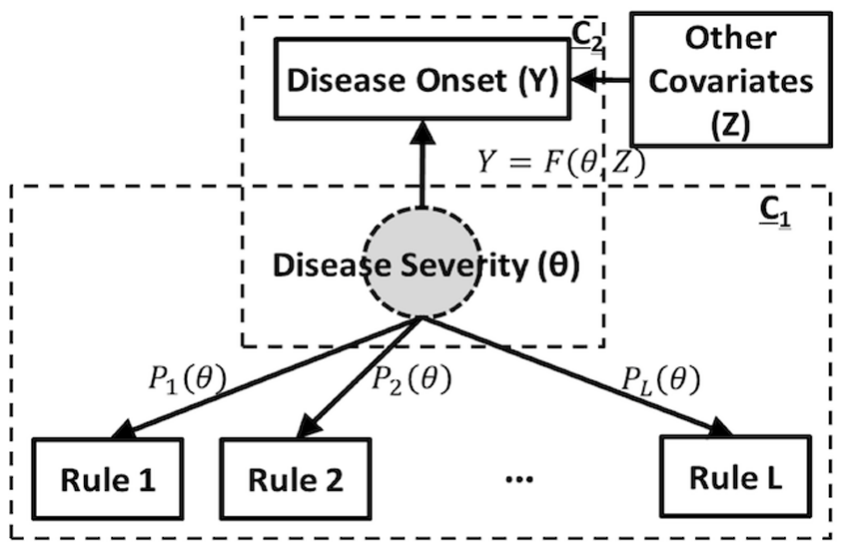
Latent trait model for rule synthesis.

As traditional latent trait models can only be used to estimate the associations between the discovered rules with the underlying disease severity, in this paper, we develop a novel latent trait model which is not only capable of modeling these associations (as shown in the box labeled with C_1_ in Fig. 1), but alsopredicting the disease onset (as shown in the box labeled with C_2_ in Fig. 1). With these associations, the likelihood of endorsement of each rule can be calculated and the latent trait model will further capitalize on these likelihoods to infer the underlying disease severity and predict the disease onset.

To model the association between the disease severity with disease onset, the latent trait model assumes that the probability of an individual's endorsement of a rule is a function of both the individual's (latent) disease severity and the association between this rule and the disease severity, which corresponds to the information about where the rule stands in the disease severity continuum and how predictive the rule is. In practice, this relationship is modeled by a monotonically increasing function called the item characteristic curve (ICC). For example, the two-parameter logistic model (2PL) can be specified for rule 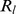 as follows:
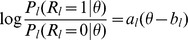



Here,
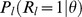
on the left hand side is the probability of an endorsement of the rule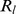, given the (latent) disease severity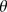. On the right side, 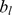
is the *item difficulty* parameter for rule 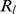 that determines the position of the ICC in relation to the disease severity scale. The item difficulty is the disease severity level required to achieve a 50% change of an endorsement, i.e.,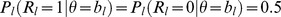. As 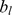 increases, the item becomes more useful to identify subjects with higher disease severity. For example, if a subject endorses a symptom, 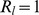, that has a large 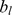, then it is unlikely that the disease severity 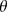
is small, since 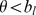
means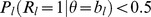. The smaller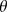, the smaller probability of observing 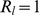, which contradicts with the data. The remaining parameter, 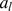, is the *item discrimination* for rule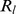, which determines the amount of change in the log odds, 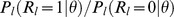, for one unit of change in the disease severity. Thus, a larger 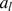
means that the rule is more sensitive to small changes in the disease severity, discriminates more clearly among the subjects, and hence is more informative and reliable. Throughout this paper, we use this two-parameter item response function due to its flexibility and interpretability. We also would like to point out that our methodology is generic and can be extended to other item response functions when needed.

It is worthy of mentioning that, the item information curve (IIC) can be extracted from the ICC, which reflects how much information a rule may have on measuring the underlying disease severity [16]. This can be achieved through the derivation of the fisher information from the 2PL model, as the fisher information reflects the information about an unknown parameter [16]. The IIC for the 2PL model is:



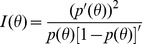
,

where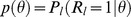 and 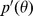 denotes the first derivative of 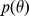
with respect to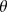.

Another task is to model the association between the disease severity with disease onset, i.e., as denoted as 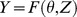 in C_2_ of Fig. 1, where 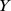
denotes the disease onset, 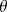
is the disease severity, and
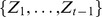
denote some other potential predictors which may provide useful supplementary information besides the rules. We can use the logistic regression model.E.g.,



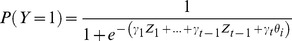
.

For instance, one exemplary predictor that can be included in 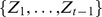 is the clinician's subject assessment of the subject's disease risk, which may provide valuable baseline assessment.

We have developed a MCMC (Markov Chain Monte Carlo) algorithm for the model parameter estimation for this latent variable model. Denote all the parameters as 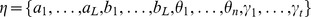 and 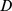 as the whole dataset that includes all the individual's measurements on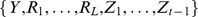. We estimate the unknown parameters 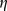
by maximizing its posterior distribution,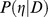, whose explicit expression can be found in the Appendix S1. The MCMC algorithm provides a computational method to estimate the posterior distribution, 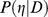, which is a description of the probabilities of possible values for 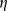
given the observed data. The MCMC algorithm is actually a sampling algorithm which draws samples from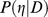. Point estimates of the parameters,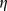, can then be obtained using statistics, e.g., mean and mode, of the posterior samples of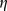. A detailed implementation procedure is given in the Appendix S1.

## Results

### 1. Identified risk-predictive rules on the DPT-1 population

RuleFit has been applied on the previously described DPT-1 dataset to derive a set of risk-predictive rules. There is no need to standardize or transform the original dataset, since RuleFit uses random forest to generate the rules, which is able to handle dataset where variables have different scales. It also has an established procedure that adapts to missing data by use of surrogate measures. Specifically, we use the baseline characteristics in DPT-1, focusing on immunologic and metabolic markers. As shown in Section 2.1, these markers include ICA, IAA, GAD, ICA 512, MIAA, fasting glucose, HbA1c, fasting insulin, first-phase insulin response (FPIR), Homeostasis model assessment of insulin resistance (HOMA-IR), and 2 hr glucose, fasting glucose, C-Peptide measurements. We have computed peak C-peptide as the maximum point of all measurements, the timing of this peak C-peptide, the early C-peptide response (30-0 min C-peptide difference) and AUC (area under curve) C-peptide using the trapezoid rule. In addition, we also include age, gender and Body Mass Index (BMI) into our model. Rules are derived on these markers by running RuleFit. By tuning the parameters using cross-validation as suggested in [11], the significant rules identified by the RuleFit model are shown in Table 2. Note that the support of a rule is the proportion of the subjects in the cohort who endorse this rule.

**Table 2:** The TOP 10 rules identified by RuleFit.

**Rule 1 (support = 33.75%)**	**Rule 6 (support = 8%)**
	24.5 (0)< FPIR <56.5 (0)
Early C-Peptide Response < 3.9 (0.67)	Peak C-Peptide < 4.75 (0.85)
	Timing of the Peak C-Peptide > 2.5 (0.12)
**Rule 2 (support = 46.88%)**	**Rule 7 (support = 57.19%)**
ICA <240 (0)	IAA < 369.7 (3.84)
IAA < 369.7 (4.84)	Fasting Glucose (IVGTT) < 103.5 (2.24)
Fasting Glucose (IVGTT) < 98.5 (1.72)	
**Rule 3 (support = 16.56%)**	**Rule 8 (support = 32.81%)**
Age < 13.89 (1.45)	ICA > 120 (3.35)
BMI > 19.27 (1.37)	AUC C-Peptide < 638.2 (3.61)
2 hr Glucose > 97.5 (4.08)	
**Rule 4 (support = 59.38%)**	**Rule 9 (support = 40.94%)**
Age < 18.24 (0)	2 hr Glucose < 117.5 (2.27)
2 hr Glucose > 87.5 (1.96)	FPIR > 70.5 (1.13)
ICA > 30 (0)	
**Rule 5 (support = 66.25%)**	**Rule 10 (support = 31.25%)**
ICA > 30 (0)	ICA > 60 (1.39)
FPIR < 155 (0)	Early C-Peptide Response < 4.1 (0.67)
7.906 (1.04) < Age < 18.24 (0)	

Note: the value in the bracket indicates the standard derivation that is calculated by the 80/20 cross validation as described in Section 3.4.

### 2. Validation of the identified rules by survival analysis

**Figure 2:**
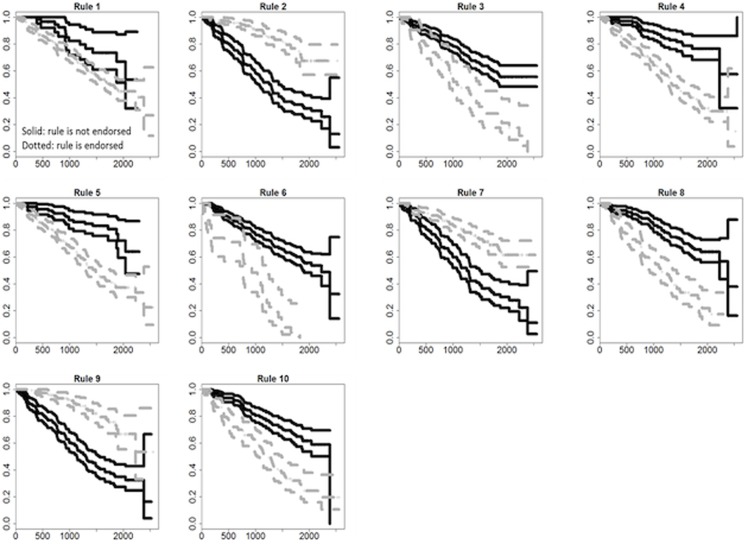
Kaplan-Meier survival curves (with their 95% confidence intervals) of the two groups defined by each rule: one satisfies the rule (dotted curve) and one doesn't (solid curve).

As each derived rule defines two groups, one satisfying the rule and the other one not, survival analysis can be applied to evaluate the separation of these two groups. Therefore, to test the prognostic values of these ten rules, we have performed the Kaplan-Meier survival analysis on the same 356 subjects for each of the top 10 rules. The results are shown in Fig. 2. In each plot, the solid curve represents the survival curve together with their 95% confidence intervals of the group for which the rule is not endorsed. The dotted curve represents the group for which the rule is endorsed. We show the p-value(i.e., logrank test) of the group separation of each rule in Table 3. It is apparent that all the rules are significant based on both Kaplan-Meier analysis and logrank test.

**Table 3:** P-values of the logrank test of the ten rules.

Rules	P-value of the Logrank test	Rules	P-value of the Logrank test
Rule 1	0.0091	Rule 6	4.44e–15
Rule 2	1.98e–13	Rule 7	8.95e–10
Rule 3	2.36e–07	Rule 8	1.12e–12
Rule 4	3.5e–10	Rule 9	3.55e–11
Rule 5	1.13e–07	Rule 10	1.73e–09

### 3. Modeling the associations between the rules with the underlying disease risk

**Figure 3:**
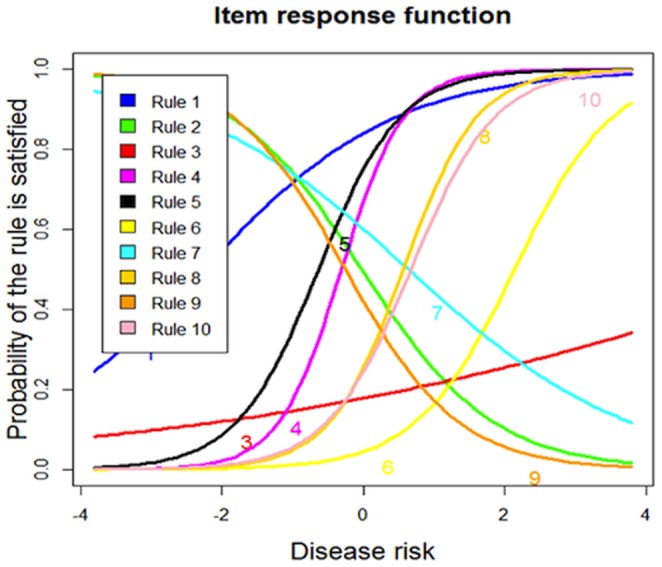
Item response functions of the 10 rules.

**Figure 4:**
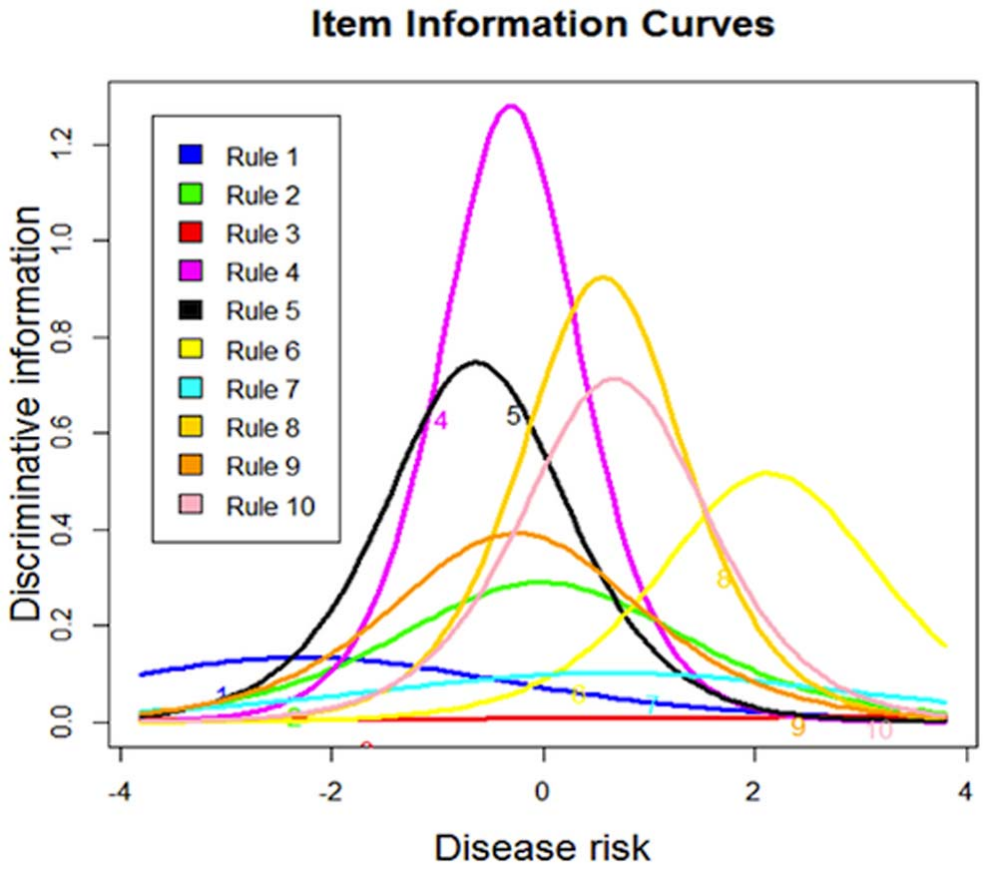
Item information curves of the 10 rules.

We have implemented the MCMC algorithm described in the Appendix S1 to fit the parameters of the item response functions in WINBUGS [17]. We draw N = 25000 samples and discard the initial 5000 samples generated by the MCMC algorithm for warming-up[17]. Convergence of the MCMC algorithm is checked according to the guidance provided in [17], e.g., by comparing the samples obtained from several runs. The means of the posterior samples of 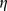 are used as the parameter estimations. With the estimated 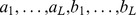, the curves of the fitted item response functions can be obtained, which are shown in Fig. 3. We also report the information curves of the rules in Fig. 4, which reveal information about within which segment of the disease risk continuum the rules are most discriminant. From Fig. 3, it can be seen that there are two kinds of rules, one is “risk-increasing” as the satisfaction of the rules increases the disease risk; the other one is “risk-decreasing” as the satisfaction of the rules decreases disease risk. It is also clear that the relationship between the rules with disease risk is very different. For example, it can be seen from Fig. 3 that, when disease risk is low to moderate, rules 4 and 5 are likely to be satisfied. On the contrary, rule 6 is not very likely to be satisfied until the disease risk is high. This implies that if a subject endorses rule 6, it is very likely the disease risk is high. The item information curve of rule 6 in Fig. 4 also demonstrates that rule 6 is the most informative item on the high-risk segment of the disease risk continuum. We also observe that the item response functions of some rules are very similar, such as rule 4 with rule 5, rule 8 and rule 10.

### 4. Assessment of the prediction accuracy of the proposed rule-based method

As mentioned in previous sections, unlike existing risk score models [6]–[8] that are only capable of stratifying subjects into different risk levels, our proposed rule-based prognostic method can assess individual's risk and predict the disease onset. Thus, it is of interest to investigate the risk prediction ability of the proposed latent trait method. We use an 80/20 validation procedure to evaluate the performance. Within this cross-validation procedure, the data is randomly divided into a training set (with 80% of the whole samples) and a testing set (with 20% of the whole samples). Rather than using the 10 rules (listed in Table 2) that have been identified on the whole dataset to fit the prediction model, here, to avoid overstatement of the prediction performance, we use RuleFit on the training set to identify the rules. Afterward, again, the latent trait theory is applied to these rules, fitted on the training data and gives classification accuracy on the testing data. Since at this stage, we don't have prior knowledge on which covariates should be included as
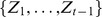
in the classification model, we only use the latent disease severity 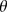 as the predictor. This 80/20 validation procedure is repeated 100 times and 100 pairs of AUC values can be obtained. The average area under curve (AUC) value is 0.82. For comparison, we also have applied the decision tree, random forest, logistic regression, SVM (Support Vector Machine)with linear kernel, Gaussian kernel, polynomial kernel, on the original variables, but the average AUCs are only 0.71, 0.74, 0.62, 0.67, 0.65, 0.58, respectively. Note that the parameters used in these models are tuned according to the standard 10-fold cross validation procedure with a grid search. For example, in tuning the parameters of the SVM with linear kernel, the only parameter is the soft margin parameter, while the parameters of the SVM with Gaussian kernel has one more parameter, the kernel parameter. In the search of the best combination of the kernel parameter and the soft margin parameter, a grid search with exponentially growing sequences is usually used, e.g., in our case,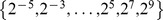 and
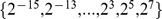
for the two parameters, respectively. Each combination of the parameter choices is evaluated using the 10-fold cross validation procedure, and the combination with the best cross-validation accuracy is picked. All the models for comparison are implemented using the routines in MATLAB.

We'd like to mention that the rules identified by the Rulefit in the training datasets are quite consistent with the 10 rules listed in Table 2. All of them frequently appear in the selected rules on each training dataset, while only small variations on the cut-off values are observed on some rules as shown in Table 2.

Furthermore, it is of interest to evaluate the effectiveness of the proposed rule-based method on other datasets rather than the DPT-1 baseline data. Six datasets (sonar, liver, pima, breast cancer, appendicitis, heart) are chosen from the UCI machine learning data repository that have similar dimensionality and sample size as the DPT-1 baseline data. We also simulate a dataset using the following strategy: First, we fit a latent trait model of the ten rules (as shown in Table 2) using all the samples in the DPT-1 dataset. Then, we randomly generate samples from this latent trait model, i.e., by randomly generating the values of the ten rules and then generating the value of the disease onset using the probabilistic relationships between the rules with the disease onset. Note that we generate the same number of samples as in the DPT-1 dataset. Finally, the same 80/20 cross-validation procedure is applied on each of the datasets including the six UCI datasets and the simulated dataset. The prediction performances (measured by the AUC values) of all the methods on all these datasets are shown in Table 4, which clearly demonstrate that the proposed rule-based method outperforms other competing algorithms across all the datasets.

**Table 4:** Prediction performances of different methods.

	Sonar	Liver	Pima	Cancer	Appendicitis	Heart	Simulated	DPT-1
Rule-based	0.89	0.74	0.75	0.95	0.94	0.86	0.94	0.82
Decision tree	0.72	0.65	0.69	0.90	0.91	0.78	0.76	0.71
Random forest	0.84	0.71	0.76	0.95	0.93	0.82	0.88	0.74
SVM (linear)	0.82	0.68	0.72	0.93	0.87	0.79	0.65	0.62
SVM (Gaussian)	0.84	0.64	0.68	0.94	0.89	0.76	0.67	0.67
SVM (Polynomial)	0.84	0.65	0.71	0.88	0.91	0.75	0.65	0.65
Logistic regression	0.76	0.67	0.65	0.92	0.89	0.77	0.55	0.58

## Discussion

The comprehensive baseline data from DPT-1 provides us the opportunity to identify some predictable patterns from genetic, immunologic, and metabolic markers, which can be used to separate out underlying disease risk, identify progression types, and pinpoint the progression stage. Using a unified framework of RuleFit and latent trait theory, several risk-predictive rules are identified, and its risk estimation performance is examined by cross validation. The results in our study suggest that the ten rules in Table 2, together with the latent trait theory that synthesizes the information of these rules, provide a good risk estimation model.

The important risk factors that are significantly involved in the ten rules in Table 2 are ICA, age, 2 hr glucose, IAA, FPIR, fasting glucose, BMI, and some C-Peptide markers. Among them, ICA, IAA, GAD, are autoantibodies, which have been found to be associated with T1D in a number of studies in literature [2], [6]–[8], [12], [18]. Existing evidences also show that FPIR, C-Peptide markers, 2 hr glucose, and fasting glucose are important in the development of risk score models [12], [6], [7]. Age and BMI have also been reported to be predictive of T1D in DPT-1 [6], [7] and some other cohorts [19]. Our results also show that gender is found to be significantly interacting with the other variables included in our study. This is consistent with previous findings that incidence trends generally do not differ between genders [20].

In these derived rules by RuleFit, the cutoff points of the markers in the rules are consistent with reported results in existing studies. For example, the studies in [21], [22] classified subjects into three risk categories according to FPIR: low risk group if FPIR > 80–100 mU/L, intermediate risk group if 80 mU/L > FPIR > 65 mU/L, high risk group if FPIR < 65 mU/L. Similar results were also shown by the study conducted by the Islet Cell Antibody Registered Users Group in [21], which classified subjects into three risk categories according to FPIR: low risk group if FPIR > 100 mU/L, intermediate risk group if 100 mU/L > FPIR > 50 mU/L, high risk group if FPIR < 50 mU/L. Our results are similar with theirs: i.e., in Rule 6, FPIR < 56.5 mU/L is considered as a sign of high risk which is close to the cutoff point, 65 mU/L; In Rule 9, FPIR > 70.5 mU/L is considered as low risk, which is close to the cutoff point, 80–100 mU/L. The slight differences between the cutoff points in our study with theirs may be due to the fact that both Rule 6 and Rule 9 involve interactions of FPIR with other markers.

The 2 hr glucose level is involved in Rules 4, 8, and 9. Among these three rules, Rules 4 and 8 are risk-increasing rule, while Rule 9 is a risk-decreasing rule. According to 1999 WHO diabetes criteria, the normal range of 2 hr glucose level is < 140 mg/dL, the impaired glucose tolerance is >140 mg/dL, and the diabetes mellitus is >200 mg/dL. The cutoff point in Rule 9 is consistent with these criteria, as 2 hr glucose < 117.5 mg/dL is not considered a risk by the 1999 WHO diabetes criteria as well. An interesting discovery is that the cutoff points in Rules 4 and 8 are 87.5 mg/dL and 97.5 mg/dL, respectively, beyond which the subject is predicted with increased risk. As 87.5 mg/dL or 97.5 mg/dL are within the normal range, our results indicate that even the 2 hr glucose is in its normal range, it is still possible that it will be indicative of T1D risk. A similar result was reported (that was also conducted on DPT-1 cohort), which revealed to reveal that the 2 hr glucose > 114 mg/dL is the optimal cutoff point for classifying the progressor from nonprogressor. A few studies [10] have shown that some markers within their conventionally defined normal ranges may still be predictive of disease risk. One explanation is that, in Rules 4 and 8, the 2 hr glucose level is considered in conjunction with Age, ICA and C-Peptide, i.e., ICA > 30 JDF Unit and ICA > 120 JDF Unit have been derived in Rules 4 and 8, respectively. As ICA > 10 JDF Unit is usually considered as evidence of risk, e.g., that was used in DPT-1 protocol, a larger ICA value may push the normal range of 2 hr glucose to a smaller value than 140 mg/dL. Existing research in literature has shown that the cutoff point of one marker may depend on some other markers. For example, in [23], authors stated that the cutoff point of HOMR-IR depends on BMI, i.e., HOMA-IR > 4.65 if BMI > 28.9 kg/m^2^, or HOMA-IR > 3.6 if BMI > 27.5 kg/m^2^; both cut-offs are indicative of insulin resistance.

The fasting glucose level derived from IVGTT is considered as risk-decreasing in Rules 2 and 7, if IVGTT < 98.5 mg/dL and IVGTT < 103.5 mg/dL, respectively. These cutoff points are similar with existing studies [10], where IVGTT < 96 mg/dL was definedwithin the normal range. However, the interpretation of Rules 2 and 7 needs extra caution, since ICA < 240 is involved in Rule 2 and IAA < 369.7 is involved in Rule 7. As ICA > 10 JDF Unit and IAA > 80 nU/mL [4], [5], [24] (or IAA > 39 nU/mL [25]) are usually considered as evidence of risk, there seems a contradiction. One explanation is that IVGTT < 98.5 mg/dL and IVGTT < 103.5 mg/dL are risk-decreasing evidence, which outweigh the risk-increasing evidence, ICA < 240 and IAA < 369.7. As such, these two rules maybe only statistically meaningful. The clinical underpinning of these two rules needs to be further investigated, since the support of these two rules are as high as 46.88% and 57.19%, respectively, which indicates that a considerable proportion of subjects in DPT-1 cohort express these patterns.

The AUC C-Peptide and Peak values of C-Peptide are involved in Rules 6 and 8. In our study, the observation that AUC C-Peptide < 638.2 indicates risk is consistent with a previous study on the DPT-1 cohort [10], which showed that AUC C-Peptide < 595 indicated risk. The Peak value of C-Peptide < 4.75 is also close to the reported result in [10], in which Peak value of C-Peptide < 5.3. In addition, we also find that the timing of the Peak C-Peptide and the early C-Peptide response are both important risk factors. Particularly, as early C-Peptide response is related to insulin secretion, the cutoff point used in Rule 1, as low as 3.9, indicates loss of insulin secretion. Also, both Age and BMI are two common risk factors in predicting T1D, such as the risk score models in [6], [7]. It is interesting to observe that these two risk factors form a single rule, Rule 3, without interacting with another risk factor.

The latent trait theory has also revealed a considerable heterogeneity of the relationships between the rules with the underlying disease risk, indicating that the biological underpinnings of these rules maybe quite different. It successfully distinguishes the risk-increasing rules from the risk-decreasing rules, i.e., Rules 2, 7 and 9, which are consistent with the Kaplan-Meier survival analysis as shown in Fig. 2. From Figs. 3 and 4, it can be seen that the ten rules provide a pretty good coverage of the middle part of the disease risk continuum, i.e., the moderate-risk part, but less informative at higher levels of disease risk, and least informative at lower levels of disease risk. This indicates that the ten rules may lead to accurate risk estimation on the subjects that have moderate-risk of developing T1D, with smaller statistical error than the risk estimation of low-risk or high-risk group. If more accurate estimation is needed for ascertaining how low or how high the risk is, more rules that cover these two groups are needed.

By utilizing the relationships between rules with the underlying disease risk, the prediction results in Section 3.4 have demonstrated that the latent trait theory is capable of providing accurate assessment of disease risk. In summary, these results show that the latent trait theory is a powerful model that can be used to identify the roles of the rules, model the relationships between rules with the underlying disease risk, and synthesize the rules for an overall personalized risk estimation.

There are limitations of this study. First, note that the rules are identified from the DPT-1 cohort. The conclusions are contingent upon the presence of ICA, since ICA positivity is used as the inclusion criteria in DPT-1. As such, further validation on some other cohorts is needed to investigate to what extent the identified rules and the corresponding risk estimation model can be applied to other populations. Second, the rules and the corresponding risk estimation model may not be fully applicable if the methodologies for glucose and C-Peptide measurements are different from those used in DPT-1. Third, the proposed statistical methodology can be further improved to incorporate domain knowledge, since it is developed on pure statistical considerations. Therefore, some rules that are indeed predictive and clinically significant may be missed since they will not add extra predictive capability to the existing pool of rules. A more intelligent rule pruning method may be developed. It is also of interest to develop more flexible latent trait models, e.g., nonparametric latent trait models, which can allow us to make more flexible assumptions about the item response functions. Therefore, better prediction accuracy maybe obtained from such nonparametric latent trait models.

As far as we know, our work is the first personalized risk model that is ever developed for T1D, as existing risk scores models [6]–[8] are only capable of stratifying subjects into different risk levels. By identifying a comprehensive set of risk-predictive rules from data, which act as a set of sensors dispersed over the whole course of disease progression, the risk estimation can be performed by looking into each individual's profile of abnormalities. The unified statistical framework we proposed has several advantages over many existing risk score models. First, it can deal with the heterogeneity of the T1D population. Second, it can deal with a mix of nominal, ordinal, count or continuous variables; it can also combine a mixture of variables of different biological nature without interpretation difficulty, as rules can provide a clear representation of complex data. Third, as rules are scale independent, data do not need to be standardized. Fourth, the rules can be associated with the underlying disease progression by the latent variable model, leading to nice interpretation and new knowledge for clinical decision making that is not provided by existing risk score models.

It may further increase the predictive capability if more markers and clinical variables can be included in the model. Another approach is extending it to longitudinal data to incorporate predictive patterns based on the change of some markers over time. While the results in this study seem promising, we do not suggest this is the final model to be applied in clinical practice, given that the underlying biological contents of the rules are not delineated. It may also be of interest to evaluate the predictive performance of this model on some surrogate end-points, such as the appearance of autoantibodies. In conclusion, the unified framework proposed in this study is shown to be a promising tool to identify risk-predictive rules from baseline characteristic data, while the prognostic values of the rules are demonstrated by both survival analysis and latent trait theory. The identified rules, together with the latent trait theory that synthesizes the information of these rules, can be used to identify individuals at risk and monitor T1D progression. Last but not least, although the MCMC algorithm is efficient in our case since both the number of variables and the sample size are not large, as it has been known that the MCMC algorithm is usually computational demanding, particularly on high-dimensional problems, we will explore approaches for accelerating the MCMC algorithm on high-dimensional problems in our future study.

## Supporting Information

Appendix S1 **The MCMC algorithm for estimating the parameters of the latent trait model.**
(DOCX)
