# Correction: A Rule-Based Prognostic Model for Type 1 Diabetes by Identifying and Synthesizing Baseline Profile Patterns

**DOI:** 10.1371/journal.pone.0109514

**Published:** 2014-09-26

**Authors:** The PLOS ONE Staff

The DPT-1 Study Group is not included in the author byline. They should be listed as the seventh author. The full membership of the study group can be found in Appendix S2 below.

There are errors in the Funding section. The correct funding information is as follows: This work has been supported by the University of South Florida and funded by the National Institutes of Health (NIH) through the National Institute of Diabetes and Digestive and Kidney Diseases (NIDDK), the National Institute of Allergy and Infectious Diseases (NIAID), the Eunice Kennedy Shiver National Institute of Child Health and Human Development (NICHD, the National Center for Research Resources (NCRR), the National Center on Minority Health and Health Disparities (NCMHD), the Office of Research on Women’s Health (ORWH), the Juvenile Diabetes Research Foundation (JDRF); and the American Diabetes Association (ADA). The NIH grant numbers which supported this research are: DK060782, DK060916, DK060987, DK061010, DK061029, DK061030, DK061034, DK061035, DK061036, DK061037, DK061038, DK061040, DK061041, DK061042, DK061058, DK061055. The funders had no role in study design, data collection and analysis, decision to publish, or preparation of the manuscript.

## 

Appendix S2 **DPT-1 Study Group Investigators (2005).**
(DOCX)
