# Supplementary material for: A Rule-Based Prognostic Model for Type 1 Diabetes by Identifying and Synthesizing Baseline Profile Patterns
Source: PLoS One. 2014 Jun 13;9(6):e91095. doi: 10.1371/journal.pone.0091095 (PMC4057076; doi:10.1371/journal.pone.0091095)
Supplement: Appendix S1 — The MCMC algorithm for estimating the parameters of the latent trait model. (DOCX) [file pone.0091095.s001.docx]

**Appendix S1: The MCMC algorithm for estimating the parameters of the latent trait model**

Here we adopt the MCMC algorithm [17] to draw samples from$P\left(\eta|D\right)$. Based on the Bayes’ theorem,

$P\left(\eta|D\right)\propto P\left(D|\eta\right)P\left(\eta\right)$,

where$P\left(D|\eta\right)$ is the likelihood of observing the data given certain values of the parameters, i.e.,

$P\left(D|\eta\right)=\prod_{i=1}^{n} \left[\prod_{l=1}^{L} P_{l}\left(r_{il}|a_{1},\ldots,a_{L},b_{1},\ldots,b_{L},\theta_{i}\right)\times P\left(y_{i}|\gamma_{1},\ldots,\gamma_{t},Z_{1},\ldots,Z_{t-1},\theta_{i}\right)\right]$,

Here, $r_{il}$denotes the value of$R_{l}$; and $y_{i}$denotes the value of$Y$, for the $i^{th}$individual. $P_{l}\left(r_{il}|a_{1},\ldots,a_{L},b_{1},\ldots,b_{L},\theta_{i}\right)$ and$P\left(y_{i}|\gamma_{1},\ldots,\gamma_{t},Z_{1},\ldots,Z_{t-1},\theta_{i}\right)$ denote the likelihood function of the item response theory with the 2PL response function [16] and thelikelihood function of the logistic regression [20], respectively, whose mathematical expressions can be found in [16,20]. $P\left(\eta\right)$is the prior distribution for the parameters. In our case, as we have no prior knowledge of the parameters, we assume uninformative prior distributions for$\eta$by assuming each parameter is distributed as a normal distribution with mean being zero and variance being 10, e.g., $P(\gamma_{i}^{*})=N(0,10)$. The details of our MCMC algorithm are shown in Fig. 5.
