# Supplementary material for: Correction: A Rule-Based Prognostic Model for Type 1 Diabetes by Identifying and Synthesizing Baseline Profile Patterns
Source: PLoS One. 2014 Sep 26;9(9):e109514. doi: 10.1371/journal.pone.0109514 (PMC4178246; doi:10.1371/journal.pone.0109514)
Supplement: Appendix S2 — DPT-1 Study Group Investigators (2005). (DOCX) [file pone.0109514.s001.docx]

**The Diabetes Prevention Trial - Type 1 (DPT-1) Study Group:**

The **DPT-1 Steering Committee** included: Jay S. Skyler, M.D. (University of Miami) (Chair), David Brown, M.D. (University of Minnesota), H. Peter Chase, M.D. (Barbara Davis Center for Childhood Diabetes, University of Colorado), Elaine Collier, M.D. (NIAID), Catherine Cowie, Ph.D. (NIDDK), George S. Eisenbarth, M.D. (Barbara Davis Center for Childhood Diabetes, University of Colorado), Judith Fradkin, M.D. (NIDDK), Gilman Grave, M.D. (NICHD), Carla Greenbaum, M.D. (Benaroya Research Institute, Seattle), Richard A. Jackson, M.D. (Joslin Diabetes Center), Francine R. Kaufman, M.D. (Childrens Hospital Los Angeles), Jeffrey P. Krischer, Ph.D. (University of South Florida), Jennifer B. Marks, M.D. (University of Miami), Jerry P. Palmer, M.D. (University of Washington), Alyne Ricker, M.D. (Children’ Hospital, Boston), Desmond A. Schatz, M.D. (University of Florida), Darrell Wilson, M.D. (Stanford University), William E. Winter, M.D. (University of Florida), Joseph Wolfsdorf, M.D. (Children’ Hospital, Boston), Adina Zeidler, M.D. (University of Southern California). Previous members were: Howard Dickler, M.D., Richard C. Eastman, M.D., Noel K. Maclaren, M.D., John I. Malone, M.D., and R. Paul Robertson, M.D.

This manuscript was prepared by a **Writing And Review Committee** consisting of Jay S. Skyler, M.D., Jeffrey P. Krischer, Ph.D., Joseph Wolfsdorf, M.D., Catherine Cowie, Ph.D., Jerry P. Palmer, M.D., Carla Greenbaum, M.D., David Cuthbertson (University of South Florida), Lisa E. Rafkin-Mervis, M.S. (University of Miami), H. Peter Chase, M.D., and Ellen Leschek, M.D. (NIDDK).

The **DPT-1 Planning Committee** included: Jerry P. Palmer, M.D. (Chair), H. Peter Chase, M.D., Catherine Cowie, Ph.D., Judith Fradkin, M.D., George S. Eisenbarth, M.D., Ph.D., Carla Greenbaum, M.D., Kevan Herold, M.D. (Columbia University), Francine R. Kaufman, M.D., Jeffrey P. Krischer, Ph.D., Jennifer B. Marks, M.D., Lisa E. Rafkin-Mervis, M.S., Desmond A. Schatz, M.D., Jay S. Skyler, M.D.

**DPT-1 Trial Coordinators** included: Beenu Aneju, R.N. (Stanford University), Debbie Conboy, R.N. (Joslin Diabetes Center), Roberta Cook, R.N. (University of Florida), Mary Alice Dennis, R.N. (University of Florida), Lois Finney, R.D. (University of Minnesota), Sherrie Harris, R.N. (Barbara Davis Center for Childhood Diabetes, University of Colorado), Della Matheson, R.N. (University of Miami), Marli McCulloch-Olsen (Benaroya Research Institute, Seattle), Terry Smith, R.N. (Joslin Diabetes Center), Julie Valenzuela, R.N. (Childrens Hospital Los Angeles), Noemi Vega, R.N. (University of Southern California).

The **DPT-1 Data Safety and Quality Monitoring Committee** included: Oscar B. Crofford, M.D. (Melbourne, Arkansas), David DeMets, Ph.D. (University of Wisconsin), John M. Lachin, Ph.D. (George Washington University), Jørn Nerup, M.D. (University of Copenhagen), Aldo Rossini. M.D. (University of Massachusetts), Alicia Schiffrin, M.D. (McGill University), Michael Steffes, M.D. (University of Minnesota), Anastasios Tsiatis, Ph.D. (North Carolina State University), Bernard Zinman, M.D. (University of Toronto).

# Affiliates: Akron, OH: A. Haider, J. Haas, Albany, NY: R. Busch, K. Marshilok, N. Toleno, Albuquerque, NM: D. Schade, P. Katz, D. Hornbeck; Alexandria, LA: S. Foster, W. E. Roberts, M. Vercher; Ann Arbor, MI: C. Foster, C. Bower; Atlanta, GA: I. L. Hansen, D. Burcell, S. Anderson, R. Shultz, C. Sparks; Austin, TX: J. Wray, L. Goldman, R. M. Holt; Baltimore, MD: D. Counts, D. Ostrowski, L. Plotnick, P. Fechner, C. Donnelly; Baton Rouge, LA: P. Bourgeios, LA: P. R. Prosser, B. Bowden; Billings, MT: F. Gunville, C. McClave, P. Heldt; Birmingham, AL: F. Ovalle, J. A. Atchison, P. Trull, A. Bottomlee; Bismarck, ND:, S. Betting, T. Davis, J. Wetzstein; Boise, ID: C. Clinkingbeard, J. Davis, T. S. Roosevelt; Bronx, NY: H. Shamoon, H. Duffy; Brooklyn, NY: H. Anhalt, L. Brussard, M., J. V. Capotorto, P. Sheehan, S. A. Quyyumi, N. D. Cohen, B. Recker, S. Castells, W. Bastian, T. W. AvRuskin, V. Verdia; Buffalo, NY: T. Quattrin, K. Dwigun; Burlington, VT: W. Cefalu, N. Clark, L. Tilton; Calgary, Alberta: B. Corenblum, S. Harries, A. Whitty; Camp Hill, PA: R. McInroy, S. Smith; Charleston, SC: S. M. Willi, L. A. Key, Jr., D. S. O’Rear, S. M. Willi, D. S. O'Rear; Charleston, WV: S. R. Grubb, K. Taylor, P, Adams; Chattanooga, TN: M. Reeves, P. Reeves, R. Marshall, E. Tessmann; Chicago Heights, IL: A. Dwarakanathan, C. Beebe, I. Weintraub-Yohay, P O'Donnell; Chicago, IL: B. Rich, J. Imperial, B. Silverman, D. Edidin, S. Goodman, I. Brodsky, L. Brodsky; Cincinnati, OH: D. Klein, L. Dolan, D. Standiford; Cleveland, OH: D. Rogers, C. Switzer; Columbia, MO: D. E. Goldstein, D. Eichelberger, A. Smith; Columbus, OH: C. Ganong, J. GermAK: W. B. Zipf, M. Dyas, J. F. Sotos, C. Young; Corpus Christi, TX: W. Riley, S. Salai; Dallas, TX: P. Raskin, J. Marks, M. Alford, R. Sachson, C. Lebowitz; Des Moines, IA: J. Cook,J. Stedman, D. Indra; Detroit, MI: J. Gutai, B. Vinuya, M. McGraw-Maly; Duarte, CA: W. Feng, C. Williams, C. M. Krygsman, M. Pierce; Durham, NC: M. Freemark, J. Litton; Edmonton, Alberta: E. A. Ryan, K. Todd; El Paso, TX: D. Aboud, M. Pacillas; Erie, PA: J. H. Hines, A. F. Walczak, L. F. Aparicio, D. Harbaugh; Fairbanks, AK: M. Bergeson, M. Rozell; Fairfield, AL: E. Mahan; Folcroft, PA: H. Brooks; Fresno, CA: P. Ginier, P. Hensley; Glen Ellyn, IL: M. Heymann, B. Johnson, J. Tack; Grand Forks, ND: L. Sondrol, T. Hjelle; Grand Rapids, MI: D. Perry, J. Albert, L. Flory; Great Falls, MT: N. C. Gerrity, C. Naab, J. Heck; Greenville, SC: S. Weber, P. Mulhall; Gulfport, MS: B. G. Lansden; Halifax, Nova Scotia: E. Cummings, S. Salisbury, C. A. Armour; Hartford, CT: S. Ratzan, M. Trahiotis; Hollywood, FL: R. Nemery, H. Carney, D. Shorkey; Honolulu, HI: D. Fitz-Patrick, A. M. Y. Taniguchi; Houston, TX: S. Gunn, K. Copeland, S. McGirk; Idaho Falls, ID: J. Liljenquist, C. Fielding, S. Richards, V. Best; Indianapolis, IN: H. Rodriguez, G. Freidenberg, L. Amstutz, C. Weir; Iowa City, IA: E. Tsalikian, R. Hoffman, M. Bayless; Kalamazoo, MI: J. D. Hare, K. Hare; Kansas City, KS: J. L. Kyner, G. Eaks; Kansas City, MO: C. P. Howard, W. Moore, B. Woodford, T. Salyer; Kennewick, WA: N. Wannarachue, R. Meridith, D. Squires; Kiel, WI: D. Deubler; Kingsville, TX: H. Bruschetta; Knoxville, TN: D. A. Nickels, C. Dothard, A. Courtney; Laguna Hills, CA: A. O. Marcus; Lebanon, NH: P., J. Beisswenger, S. Kairys, W. Boyle, A. S. Christiano, R. O’Dell, A. Touchette; Lexington, KY: K. M. Thrailkill, D. Karounos, S. Webb, L. Moore, P. Allweiss, F. Anderson ; London, Ontario: J. L. Mahon, J. McCallum; Los Angeles, CA: L. Raffel, J. Rotter, A. Verne, M. B. Davidson, G. Keppler; Madera, CA: S. Banerjee, M. Simon; Madison, WI: M. J. MacDonald, S. Mokrohisky; Manhasset, NY: P. W. Speiser, P. Fort, J. Corrigan; Marquette, MI: S. Pelkola; Memphis, TN: A. E. Kitabchi, M. Murphy, H. Lambeth, G. Burghen, P. McGlendon, J. Bondani, P. LeNoye; Milwaukee, WI: R. Alemzadeh, M. Koppen; Mineola, NY: J. A. Canas, M. Lamerson; Minneapolis, MN: M. Spencer, D. Etzwiler, K. Reynolds; Missoula, MT: N. Eyler, P. Allen; Mobile, AL: B. A. Warner, K. R. Rettig, K. L. Levens, M. R. Davis; Montreal, Quebec: C. Polychronakos, D. Laforte; Naperville, IL: W. P. Zeller, J. McKernan, S. Finn; Nashville, TN: A. Powers, J. Lipps; New Albany, IN: S. Raghavan, V. Broadstone, P. Raake, K. Weissberg; New Haven, CT: W. V. Tamborlane, P. Gatcomb; New Orleans, LA: L. Blonde, T. Zimmerman, R. Zimmerman, C. Liebel, S. Chalew, A. Vargas, J. Rao, J. Ascani, T. Compton; New York, NY: B. Cerame, M. D. Harbison, Ron Newfield, MD, M. New, M. Wajnrajch, I. Vargas, K. Herold, H. Schachner, G. Feberes , N. K. Maclaren, D. Golub, R. Rappaport, R. McEvoy, N. Thomas, X. Pi-Sunyer, R. Saltiel-Berzin; Newark, NJ: R. Rappaport, J. Koblish; Oklahoma City, OK: P. Blackett, C. Comp, J. Beck; Omaha, NE: J. Hassing, L. Hahn, J. T. Lane, K. Corley, L. Larson; Orange, CA: R. Fiallo-Sharer, P. Lee, A. Cortez, N. Varni, H. Speer; Orlando, FL: S. Crockett, W. McDaniel, V. Roberts; Philadelphia, PA: S. A. Weinzimer, P. Cohen, L. Baker, D. De Paul, E. Rebecca; Phoenix, AZ: R. Dolinar, M. B. Block, P. Krametbauer; Ponce, PR: T. Frazer, G. Veray; Portland, OR: A. Ahmann, S. LaFranchi, P. Jennings, A. Kelleher, M. Kummer, J. Hansen, M. K. Hunter, K. LaMorticella, Bergstrom, M. Rigdon; Reno, NV: K. Eckert; Renton, WA: L. J. Klaff, R. Brazg, J. Springs; Richland, WA: B. Wilson, E. Isaacson, H. Kuhn; Richmond, VA: D. Willis, P. Kaplowitz, K. Genther; Rio Piedras, PR: C. Bourdony, Jr., A. Rivera; Rochester, MN: R. Basu, R. Rizza, P. Whannel, N. Jospe, A. Utzman; Sacramento, CA: B. Sheikholisham, C. Hiner; Salt Lake City, UT: D. Hardin, R. Lindsay, M. Swinyard, M. Rallison, L. Jarrett, J. Sirstins; San Antonio, TX: K. Pierson, S. Trevino, S. Schwartz, K. Dickens; San Bernardino, CA: S. Clark, P. Scroggin, G. R. Greene; San Diego, CA: L. Linarelli, E. Camuro, S. Hermosillo, R. Estrada, W. Bailey, J. Fuqua; San Francisco, CA: S. E. Gitelman, M. Fountaine; San Juan, PR: G. Colon, L. Gonzalez de Pijem, F. Nieves-Rivera, A. Rivera, R. Perez; Santa Barbara, CA: L. Jovanovic, S. Vesterfelt; Santurce, PR: C. A. Saenz; Seattle, WA: G. Kletter, K. Pihoker, S. Kearns; Sioux Falls, SD: L. Keppen, P. Johnson, E. Krell; Skokie, IL: S. C. Duck; Spokane, WA: M. Noble, S. Thompson, K. Wilson, P. ; Springfield, IL: N. G. Soler, L. McCall; Springfield, MA: H. F. Allen, G. Roumeliotis; Springfield, MO: L. Chase, D. Braden-Moll; St. Louis, MO: N. White, L. Levandoski; St. Petersburg, FL: J. I. Malone, J. Steinbrueck; State College, PA: J. S. Ulbrecht, N. Lambert, P. Mulhall; Stony Brook, NY: T. A. Wilson, A. H. Lane, A. Smaldone; Sylmar, CA: T. Modilevsky; Syracuse, NY: R. Izquierdo, R. Weinstock, S. Mackowiak, K. Brindak; Toronto, Ontario: D. Wherrett, D. Daneman, K. Pearlman, C. McLellan, A. Rogers; Torrance, CA: E. Ipp, C. Mao; Traverse City, MI: E. H. Rushovich, I. Thorne; Tulsa, OK: D. H. Jelley, D. Greer; Vancouver, BC: D. Metzger; Washington, DC: A. Austin, A. Glasgow, J. Turek, J. Archer, G. Nunlee-BlaND: K. Johnson, J. Harris; White Plains, NY: S. Driedbart, R. Noto, A. Romano, W.Herl; Wichita, KS: R. Guthrie, O. Tatpati, A. Brenner; Willmar, MN: D. Lippert; Wilmington, DE: G. Reeves, C. Swenson; Winnipeg, Mb:, L. Murphy, H. Dean, L. Berard; Winston-Salem, NC: S. S. Werbel, A. Bell-Farrow; Woodinville, WA: R. Mauseth, J. Hanson; Youngstown, OH: S. K. Mishr, L. DiCaria, B. Wilson

**Satellites**: Aberdeen, SD: C. Wischmeier; Akron, OH: M. F. Moosa, R.Levy; Albany, NY: J. Desemone; Albany, OR: L. Bentson; Alexandria, VA: H. M. Lando; Alton, IL: J. Hoelscher; Amarillo, TX: W. C. Biggs; Ames, IA: R. Carano; Anaheim, CA: P. Nostrand; Anchorage, AK: C. EsquivAL: L. Achee, J. Kelly, P. Nolan; Apple Valley, CA: T. Otsuka; Appleton, WI: K. Heyrmann; Astoria, OR: N. Autio, K. Gohl; Atherton, CA: J. Prendergast; Atlanta, GA: B. Bode, H. Delcher, C. Hamilton Reed; Atlantis, FL: M. Mellman; Augusta, GA: I. C. Herskowitz, W. Hoffman; Augusta, ME: M. Naas; Austin, TX: S. Dubois, Fehrenkamp; Aventura, FL: L. B. Chaykin; Bakersfield, CA: H. Pershadsingh, H. Shah, V. G. Ettinger; Baltimore, MD: B. J. Reiner, J. McLaughlin, P. A. Levin; Bangor, ME: A. Boniface; Bassett, NE: H. Leigh; Batavia, NY: G. Ginsberg; Bedford, OH: D. Weiss; Belleville, IL: M. Rosecan; Bellevue, WA: P. Doyle; Bellingham, WA: J. McAfee, G. Goldfogel; Bend, OR: J Henschel; Bennington, VT: D. M. Gorson; Bethehem, PA: J. Ramos; Beverly Hills, CA: M. Bush; Bismarck, ND: K. Martin; Blacksburg, VA: B. Birch; Bremerton, WA: S. Reimer; Bristol, TN: J. D. Neil; Bronx, NY: P. Saenger, J. Dimartino-Nardi; Brooklyn, NY: J. V. Capotorto, P. Sheehan, S. A. Quyyumi, N. D. Cohen; Burbank, CA: R. Stein; Burlingame, CA: D. Klonoff; Butte, MT: J. de Souza, MD: D. McCarthy, J. Salisbury, C. Edstrom; Caguas, PR: M. F. William; Caldwell, ID: M. Brown; Camp Springs, MD: R. Vigersky; Canton, OH: C. E. Smith, A. Krishna, R. Benson; Castaner, PR: F.Murphy; Cedar Rapids, IA: C. Pruchno; Chapel Hill, NC: M. Davenport; Charlottesville, VA: W. L. Clarke, M. McDuffie; Cheyenne, WY: G. Melinkovich, V. Bell; Chicago Heights, IL: A. Ravanam, W. Will; Clearwater, FL: D. Leonard; Cleveland, OH: W. Dahms; Clinton, MO: K. Scott; Columbia, SC: F. Bowyer; Columbus, GA: S. B. Leichter; Concord, CA: R. Kaplan, S. Lewis; Coopers Mills, ME: R. Miller; Corpus Cristi, TX: M. Upmanyu; Culver City, CA: N. Goldberg; Danbury, CT: R. Savino; Danville, IN: S. M. Wentworth; Danville, PA: D. R. Langdon; Davenport, IA: C. Weideman; Dayton, OH: M. Urban ; Detroit, MI: D. Transue, F. Whitehouse, J. Cara; Downey, CA: S. Shaw; Drayton, SC: W. Price; Dubuque, IA: R. Iverson; Duluth, MN: M. Slag; Durango, CO: J. Hutt; Eau Claire, WI: N. McLean, WI: R. Moore; El Paso, TX: R. Christenson; Elgin, IL: K. Valika; Englewood, CO: N. Nayak, C. A. Bloch; Englewood, NJ: L. Strom; Escondido, CA: T. S. Bailey, C. P. Varma; Eugene, OR: D. Calder, M. Bilger; Everett, WA: K. Larson, M. Papenhausen; Fairfield, CA: Y. Shlesinger; Fargo, ND: A. Kenien; Fayetteville, NC: E. Wright; Flint, MI: M. A. Jabbar; Flushing, NY: D. L. Lorber; Fort Dodge, IA: J. Berkett; Fort Smith, AR: R. P. Robinson; Fort Wayne, IN: A. Kadambi; Framingham, MA: W. Sullivan; Fremont, CA: E. Meyer; Fresno, CA: J. L. Bautista, P. C. Norwood; Ft. Lauderdale, FL: E. Biederman, S. Nassberg, L. Goscin, M. Mata, N. Thompson, R. M. Harrell, J. Cabral; Ft. Myers, FL: A. Pietri; Ft. Worth, TX: C. R. Scott, T. K. Flannery, D. B. Wilson; Gainesville, FL: B. Rogers; Glendale, CA: M. Campos, M. N. Montero; Gorham, NH: B. Beals; Grand Junction, CO: D. Mair, A. Long; Grand Rapids, MI: R. S. Rood; Green Bay, WI: J. Taylor; Greensboro, NC: R. Sevier; Greenville, NC: G. Harris, M. A. Pfeifer; Hackensack, NJ: M. Blechman, J. Giangola; Hermitage, TN: R. Creech; Hershey, PA: M. Lathrop, A. Dunaif; Hollywood, FL: G. Miceli, L. Lewy-Alterbaum, P. S. Jellinger, S. B. Novak, K. M. Gellman, S. Lerman; Honolulu, HI: S. Waxman; Houston, TX: D. J. Hamilton; Huntington, WV: H. Driscoll; Huntsville, AL: R. Schneier; Hutchinson, KS: J. L. Casey; Indianapolis, IN: P. Boyce, J. Meachum; Irving, TX: J. Milburn; Issaquah, WA: D. Pomeroy; Jackson, MS: G. Moll; Jacksonville, FL: K. Macyko, L. A. Fox, N. Mauras; Jersey City, NJ: P. Ledereich; Juneau, AK: D. Novotney; Kalispell, MT: C. Gill, B. Rossetto; Kingston, ON, R. Houlden; Klamath Falls, OR: P. Heck; La Crosse, WI: J. Korducki; La Habra, CA: J. Winston, MD, D. Geffner; La Jolla, CA: S. Edelman, MD: B. Henry; La Mesa, CA: D. Einhorn, R. Fink, E. Gold; Lake Charles, LA: R. W. (RW) Calhoun, MD; Lake Jackson, TX: J. Leidlein; Lancaster, CA: K. Arul; Lansing, MI: D. Henry; Las Vegas, NV: D. L. Donaldson; Lebanon, OR: K. Middlestadt; Lewiston, ID: L. Grande-Luke; Lincoln, NE: J. Guest, R. Wermers, B. Bells; Little Rock, AR: P. Frindik; Livingston, NJ: G. Gewirtz; Lompoc, CA: C. Blyfeld; Long Beach, CA: M. Brakin; Los Angeles, CA: D. Borut, D. Geffner, J. Winston, M. Geffner, M. Rodriguez , V. Gura, M. Rodriguez; Los Gatos, CA: C. Shough; Louisville, KY: H. Bays, H. Shenouda; Lubbock, TX: S. Varma, M. J. Bourgeios; Lufkin, TX: L. A. Sloan; Lynchburg, VA: C.E. Guthrow, Jr.; Lynwood, CA: S. Shaw; Manhattan Beach, CA: R. Ruby; Margate, FL: B. Motzkin-Kava; Marshalltown, IA: D. Jebsen; Marshfield, WI: I. Zador, S. Maby; McLean, VA: F. Crantz; Medford, OR: D. Zietlow; Miami Beach, FL: D. Kudzma; Miami, FL: E. Levy, E.T. Shapiro, J. Jacobi,J. Pita, W. Abelove, J. Perez-Rodriguez, L. Gonzalez-Mendoza, S. Richton, P. Weissman; Middletown, NY: N. Stein; Midland, TX: L. Sherman-Adcock; Milwaukee, WI: D. Fergeson, M. Jacobson, J. Sennett, R. Jain; Minneapolis, MN: D. Etzwiler, R. C. Ramsay; Minot, ND: M. Holland; Miramar, FL: S. Carrington; Missoula, MT: S. Seagraves; Modesto, CA: G. M. Yue, J. Downs-Colby; Montebello, CA: H. Flores; Montgomery, AL: S. Weinrib; Morgantwn, WV: E. Jones; Morristown, NJ: H. Starkman; Mount Vernon, WA: D. White; Mountain View, CA: L. Doberne, M. Greenfield; Muncie, IN: K. Alexander; Napa, CA: C. O’Sullivan; Naples, FL: R. Duncan; Naranja, FL: G. Barandiaran, J. Yunis, L. Nunez, V. Ramos; Nashua, NH: E. Holland; Natrona Heights, PA: W. R. Balash; Neptune, NJ: J. Sher; New Brunswick, NJ: R. Agrin; New Orleans, LA: J. Frentz; New York, NY: I. Fennoy; Newhall, CA: S. Baron; Nipomo, CA: J. Door; Norfolk, VA: A. Vinik; North Las Vegas, NV: F. Savery; Oakland, CA: F. Gareis, R. Mack, D. Devoe, Y. Fan; Oklahoma City, OK: D. Domek; Olympia, WA: D. Kelley; Onalaska, WI: T. Roberts; O'Neill, NE: B. Gutshall; Orange, CA: I. Madu, R. Poucher; Orlando, FL: M. Mengel, P. Desrosiers, R. A. Banks, B. Kopp; Pacific Grove, CA: I. Fishman; Pacific Palisades, CA: D. Geller, W. Smith; Palm Bay, FL: J. A. Duncan; Palm Beach Gardens, FL: O. Nyman, M. Vaccarello-Cruz; Parkersburg, WV: F. L. Schwartz; Pasadena, CA: O. Olambiwonnu; Paso Robles, CA: M. Ortiz; Pembrooke Pines, FL: S. Freedman; Pendleton, OR: S. Merrill; Peoria, IL: J. Wise; Philadelphia, PA: I. Rezvani, D. Doyle, P. Hale, C. Singer-Granick, W. Fore; Phoenix, AZ: A. Perelman, R. Clemmons, R. Johnsonbaugh; Pittsburgh, PA: A. R. Gonzalez; Pocatello, ID: M. Baker, C. Field, C. Shields, K. Walker; Port Huron, MI: S. Reddy, K. Pillote; Port St. Lucie, FL: M. Borchelt; Port Townsend, WA: D. Bommer; Portland, OR: N. Curosh, D. Karl, B. Phillipson; Poway, CA: W. L. Iverson; Princeton, NJ: A. Krosnick; Providence, RI, C. B. Kahn; Puyallup, WA: B. Blodgett, N. Iverson, C. Jacobson, M. Haynes, D. Moore, R. Alston, R. Eachempati; Raleigh, NC: D. Becker; Rapid City, SD: L. Weide; Red Wing, MN: M. Decker; Redding, CA: J. Greaves; Richburg, SC: M. J. Curry; Richmond, VA: J. Radcliffe; Ridgecrest, CA: V. Schauf; Riverside, CA: D. Childs; Riverside, IL: R. Crawford, G. Charnogursky; Robbinsdale, MN: M. Stesin; Rockford, IL: M. Schneider; Rockville, MD: H. W. Rodbard, M. A. Dempsey; Roseau, MN: R. Brummer; Roseburg, OR: L. Elston, D. Marseters; Rutland, VT: P. Lapp; Sacramento, CA: N. Glaser, J. S. Soeldner; Safford, AZ: V. Chaurasia; Salem, OR: R. Michaels; Salinas, CA: A. King, R. Olson; San Antonio, TX: D. Hale, M. Danney, S. S. Miller; San Carlos, CA: S. Madan; San Diego, CA: J. R. Dudl; San Francisco, CA: N. Bohannon, M. Berlund, T. Jackson; San Jose, CA: J. Kitzmiller; Santa Rosa, CA: D. Price; Savannah, GA: K. Ehsanipoor; Sayre, PA: M. R. Homan; Scarborough, ME: J. Olshan; Scottsbluff, NE: T. Sorensen; Scranton, PA: A. PerryD; Seattle, WA: G. T. Nepom, D. McCulloch, I. Hirsch, R. Kanter, N. Niles; Shattuck, OK: M. Vogiatzi; Sheboygan, WI: V. Kerpe; Shreveport, LA: R. McVie; Sioux City, IA: J. Aniszewski, T. Carroll, R. Bacon; South Bend, IN: M. Hudson, A. Simpson; South Miami, FL: M. Fili, D. Krieger; South Windsor, CT: D. Golob; Southfield, MI: J. Carney; Springfield, IL: R. Khardori; St. Cloud, MN: M. Peitso; St. Louis, MO: J. T. Lane; St. Paul, MN: H. Katz, R. Warhol; Staten Island, NY: J. Rothman; Stockton, CA: D. F. Jensen, J. Rooke; Stoughton, MA: H. Fogel, L. Hotes; Sumter, SC: U. Lilavivat; Tacoma, WA: T. Gauthier; Tallahassee, FL: L. Deeb, T. Sherradan; Tarzana, CA: N. Lavin; Tecumseh, NE: K. Shuey, MD; Toledo, OH: J. Horner; Torrance, CA: J. M. Tsao; Traverse City, MI: A. Scrogin; Trexlertown, PA: C. Greenlee; Tucson, AZ: J. Insel, M. Wheeler; Tuscaloosa, AL: T. Kamal; Tyler, TX: L. Wiertz; Union, NJ: H. Bucholtz, J. Dunn; Urbana, IL: K. Wilson; Valhalla, NY: M. Frey, R. Noto; Ventura, CA: R. Chochinov; Vincennes, IN: J. M. Bridges; Waco, TX: M. Amar; Walnut Creek, CA: R. Weinstein, T. E. Poore; Waltham, MA: S. Brink, K. Moltz; Washington, DC: D. Sobel, G. Francis, R. Sveck, J. Ramey; Waterford, MI: N. Haque; Watseka, IL: A. Villafria, R. E. Villafria, Wausau, WI: J.Madagame; Wenatchee, WA: L. Stone; West Carrollton, OH: R. Cech; West Yellowstone, MT: J. Schnelbach; White Plains, NY: S. Dreidbart, R. Noto, T. Lebinger, L. Shane; Whittier, CA: E. Reece, R. Harris, W. D. Welsh; Wilmington, NC: P. C. Whitesides, Jr.; Winter Park, FL: A. Scoma; Woodland Hills, CA: F. H. Ziel; Worcester, MA: C. Alter, P. Lock; Yakima, WA: G. Treece
